# Neuromorphic crossbar circuit with nanoscale filamentary-switching binary memristors for speech recognition

**DOI:** 10.1186/1556-276X-9-629

**Published:** 2014-11-23

**Authors:** Son Ngoc Truong, Seok-Jin Ham, Kyeong-Sik Min

**Affiliations:** 1 School of Electrical Engineering, Kookmin University, 77, Jeongneung-ro, Seongbuk-gu, Seoul 136-702, South Korea

**Keywords:** Memristors, Neuromorphic, Crossbar, Speech recognition, Filamentary switching, Binary memristors

## Abstract

In this paper, a neuromorphic crossbar circuit with binary memristors is proposed for speech recognition. The binary memristors which are based on filamentary-switching mechanism can be found more popularly and are easy to be fabricated than analog memristors that are rare in materials and need a more complicated fabrication process. Thus, we develop a neuromorphic crossbar circuit using filamentary-switching binary memristors not using interface-switching analog memristors. The proposed binary memristor crossbar can recognize five vowels with 4-bit 64 input channels. The proposed crossbar is tested by 2,500 speech samples and verified to be able to recognize 89.2% of the tested samples. From the statistical simulation, the recognition rate of the binary memristor crossbar is estimated to be degraded very little from 89.2% to 80%, though the percentage variation in memristance is increased very much from 0% to 15%. In contrast, the analog memristor crossbar loses its recognition rate significantly from 96% to 9% for the same percentage variation in memristance.

## Background

The memristors that had been mathematically predicted by Leon O. Chua in 1971 as the fourth basic circuit element [1] were experimentally found in 2008 [2]. Since the first prediction of memristors, they have been thought as a potential candidate for future neuromorphic computing systems. Among the many advantages of memristors, particularly, the nonlinear charge-flux relationship is important in mimicking synaptic plasticity of biological neuronal systems such as human brains [3-7].

In realizing memristor-based synaptic systems, a crossbar circuit that is made of only passive memristors can be thought of as the densest and simplest architecture among various synaptic circuits that have been developed previously. If a crossbar circuit is made of both memristors and selectors such as transistors and diodes, this kind of hybrid-type crossbar circuit is difficult to be stacked layer by layer. Thus, the pure crossbar circuit with only passive memristors can be a key element to implement the densest and simplest three-dimensional architecture of neuromorphic systems.

**Figure 1:**
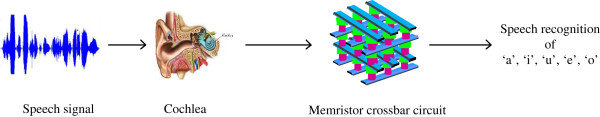
The conceptual signal flow of a neuromorphic speech-recognition system with memristor crossbar array.

A conceptual diagram of a neuromorphic speech-recognition system is shown in Figure 1. In Figure 1, a voice signal enters the cochlea first. In the cochlea, the voice input is divided into many different channels according to the voice's frequencies. Basically, the cochlea is modeled as a group of band-pass filters, where the voice input is divided and filtered by a band-pass filter array with the frequency range from 20 Hz to 20 KHz [8,9]. Each channel in the band-pass filter array can deliver a different band signal to the crossbar circuit as shown in Figure 1. Here, we assume that our goal is recognizing five vowels: ‘a’, ‘i’, ‘u’, ‘e’, and ‘o’, from the input of a human voice. To do so, the voice input is filtered and sampled as the cochlea does. Then, the filtered and sampled signals go into the memristor crossbar circuit as shown in Figure 1, where the voice input is compared with the previously trained patterns of five different vowels which are already stored in the memristor crossbar array. By doing so, we can decide which vowel among the five different vowels is the best match with the voice input to the crossbar array.

**Figure 2:**
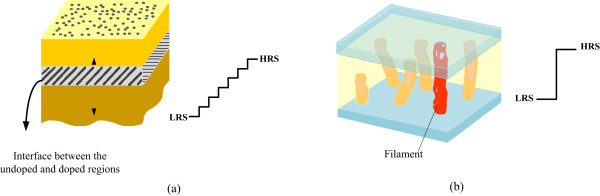
**Analog memristors with interface-switching mechanism and binary memristors with filamentary-switching mechanism. (a)** Analog memristor with the interface-switching mechanism [10,11], where the memristance value can be changed gradually from LRS to HRS, and **(b)** binary memristor with the filamentary-switching mechanism [12-17], where the memristance value can be changed very abruptly between LRS and HRS.

In realizing a memristor crossbar circuit, we can use either analog memristors [10,11] or binary memristors [12-17] as shown in Figure 2a,b. For the analog memristors in Figure 2a, their memristance value can be changed gradually and not abruptly due to the interface-switching mechanism. In the interface-switching behavior, the interface between the low-resistance region and the high-resistance region can be controlled precisely according to an applied voltage or current. As a result, we can store not only binary data but also analog data on the interface-switching memristors with high accuracy. However, materials that show the interface-switching behavior are not so popular, and the accuracy in controlling the memristance value is still considered to be a big concern. Also, even a small amount of memristance variation can degrade the overall accuracy severely in analog-memristor-based neuromorphic systems. On the contrary, most memristors are known that they are based on the filamentary-switching mechanism. In filamentary switching, memristors can have either a high resistance state (HRS) or a low resistance state (LRS) as represented in Figure 2b. By doing so, we can store only ‘1’ or ‘0’ on the filamentary-switching binary memristors.

In addition to the advantage of popularity of filamentary-switching materials, binary memristors can be much more tolerant against statistical variations compared to analog memristors. This is due to the fact that HRS can still be much higher than LRS, in spite of the large amount of statistical variation in LRS and HRS.

In this paper, we propose a binary memristor crossbar circuit for recognizing five different vowels. The block diagram and the detailed circuit schematic are shown and explained in the following section. In addition, the circuit simulation and statistical simulation are performed, and the simulation results are discussed and finally summarized in this paper [18].

## Methods

**Figure 3:**
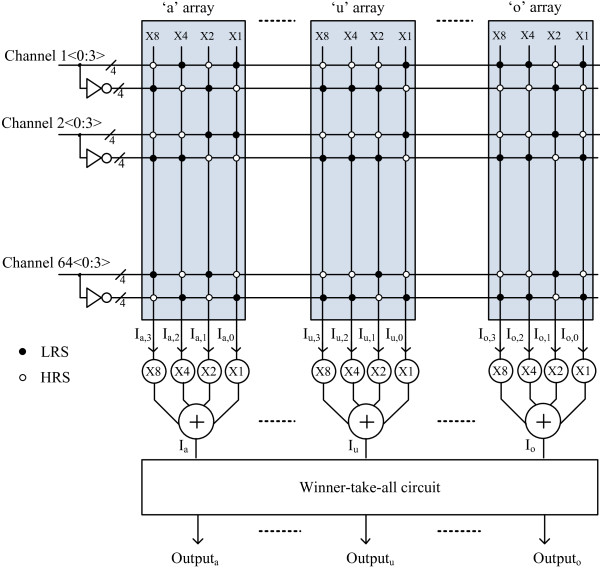
**The block diagram of the proposed binary memristor crossbar circuit with 4-bit 64 input channels.** Each 4-bit input channel is composed of the true signal and the inverted signal.

Figure 3 shows a block diagram of the binary memristor crossbar circuit for recognizing five vowels: ‘a’, ‘i’, ‘u’, ‘e’, and ‘o’. The voice input is divided into 64 channels according to the voice's frequencies. The magnitude of each channel is sampled and digitized by 4 bits. The band-pass filtering, sampling, and digitization for the voice input are implemented by MATLAB simulation in this paper. The 4-bit 64 channel inputs that are obtained by MATLAB simulation are applied to the binary memristor crossbar array as shown in Figure 3. For recognizing five vowels, we need not only 4-bit 64 channel inputs but also their inverted values. Thus, the total number of channel inputs is as many as 128 with 64 channels of the true signals and 64 channels of the inverted signals. Each channel is composed of 4-bit binary values. In Figure 3, *I*_a,0_ is the current of the ‘x1’ column in the crossbar array for recognizing ‘a’. *I*_a,1_ is the current of the ‘x2’ column in the crossbar array for recognizing ‘a’. Similarly, *I*_a,2_ and *I*_a,3_ are the currents of the ‘x4’ and ‘x8’ columns in the ‘a’ crossbar array. Here, ‘x1’ means that the weight of this column current is as much as 1. In Figure 3, ‘x2’, ‘x4’, and ‘x8’ mean that the weight values are 2, 4, and 8, respectively, for the corresponding columns in the ‘a’ crossbar array. Here, *I*_a_ can be calculated with the weighted summation of 8*I*_a,3_ + 4*I*_a,2_ + 2*I*_a,1_ + *I*_a,0_. Similarly, *I*_u_ is the weighted summation of 8*I*_u,3_ + 4*I*_u,2_ + 2*I*_u,1_ + *I*_u,0_ for recognizing ‘u’. *I*_o_ is the weighted summation of 8*I*_o,3_ + 4*I*_o,2_ + 2*I*_o,1_ + *I*_o,0_ for recognizing ‘o’. The currents of *I*_a_, *I*_i_, *I*_u_, *I*_e_, and *I*_o_ are compared with each other in the winner-take-all circuit [19] to decide which vowel is the best match with the voice input as shown in Figure 3. Output_a_, Output_i_, Output_u_, Output_e_, and Output_o_ are the output signals of the winner-take-all circuit.

**Figure 4:**
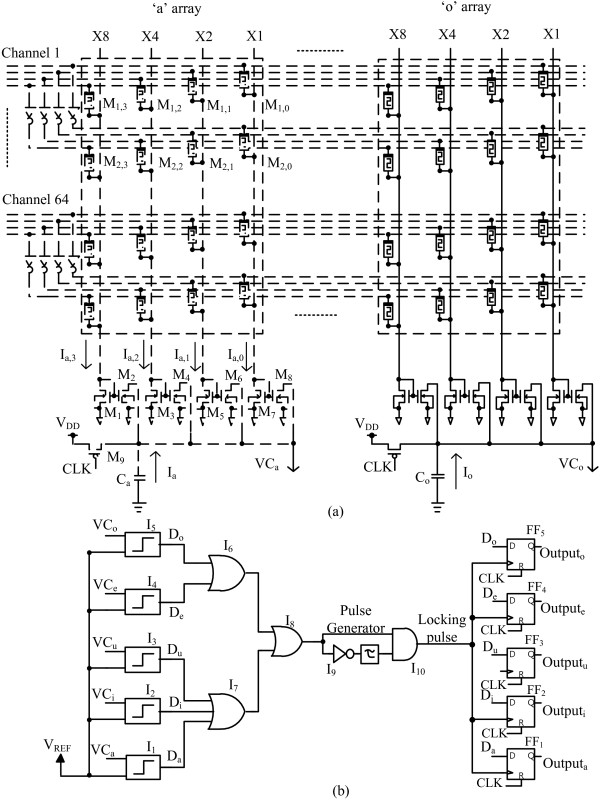
**The schematics of the binary memristor crossbar circuit and the winner-take-all circuit. (a)** The schematic of the binary memristor crossbar circuit, and **(b)** the schematic of the winner-take-all circuit.

Figure 4a shows the detailed schematic of the binary memristor crossbar circuit. Here, 64 input channels are applied to the crossbar circuit. Each channel has 4-bit binary values and each binary value is divided into true and inverted signals as shown in Figure 4a. *M*_1,0_, *M*_1,1_, *M*_1,2_, and *M*_1,3_ are memristors of the ‘x1’ column, ‘x2’ column, ‘x4’ column, and ‘x8’ column, respectively, for the crossbar array of vowel ‘a’. These four memristors are connected to the true signal of channel 1. Similarly, *M*_2,0_, *M*_2,1_, *M*_2,2_, and *M*_2,3_ are memristors of the ‘x1’ column, ‘x2’ column, ‘x4’ column, and ‘x8’ column, respectively, which are connected to the inverted signal of channel 1.

The weighted summation of *I*_a_ is calculated with 8*I*_a,3_ + 4*I*_a,2_ + 2*I*_a,1_ + *I*_a,0_, as explained just earlier. The circuit for performing the weighted summation is implemented by current mirror circuits as shown in Figure 4a. For example, to realize the weight of ‘1’, we use the current mirror circuit, which is composed of *M*_7_ and *M*_8_. Here, *M*_7_ and *M*_8_ should have the same size. By doing so, *I*_a,0_ of *M*_7_ can be copied to *M*_8_. If the weight is 2, the size of *M*_6_ should be twice larger than *M*_5_. Thereby, the current of *M*_6_ can be twice larger than *I*_a,1_. For the weight factor of 4, *M*_4_ should be four times larger than *M*_3_. For the weight factor of 8, *M*_2_ should be eight times larger than *M*_1_. The currents of *M*_2_, *M*_4_, *M*_6_, and *M*_8_ can be summated by Kirchhoff's current law. The capacitor *C*_a_ can be discharged by the weighted summation of *I*_a_, which comes from *M*_2_, *M*_4_, *M*_6_, and *M*_8_. If the weighted summation of *I*_a_ is large, *C*_a_ can be discharged to GND very fast. Here, GND means the ground potential. If the weighted summation of *I*_a_ is small, it takes longer time to discharge *C*_a_ to GND. *M*_9_ is the precharge PMOS, which becomes on when the clock (CLK) signal is low. If *M*_9_ is on, the VC_a_ node is precharged by *V*_DD_. When the CLK signal is high, *M*_9_ is off. At this time, VC_a_ can be discharged by the weighted summation of *I*_a_ that comes from *M*_2_, *M*_4_, *M*_6_, and *M*_8_.

Figure 4b shows the winner-take-all circuit that can decide which capacitor becomes discharged the fastest among the five capacitors of *C*_a_, *C*_i_, *C*_u_, *C*_e_, and *C*_o_. The five capacitors of *C*_a_, *C*_i_, *C*_u_, *C*_e_, and *C*_o_ are corresponding to the five vowels ‘a’, ‘i’, ‘u’, ‘e’, and ‘o’, respectively. Using the winner-take-all circuit, we can figure out that a certain vowel corresponding to the fastest-discharged capacitor is the best match with the input of a human voice. VC_a_, VC_i_, VC_u_, VC_e_, and VC_o_ are the voltages on capacitors *C*_a_, *C*_i_, *C*_u_, *C*_e_, and *C*_o_, respectively. Here, *I*_1_, *I*_2_, *I*_3_, *I*_4_, and *I*_5_ are the comparators. In this case, *I*_1_ compares VC_a_ with *V*_REF_. *V*_REF_ is a reference voltage to the comparators. If VC_a_ becomes lower than *V*_REF_, *D*_a_ becomes high. Similarly, *I*_2_, *I*_3_, *I*_4_, and *I*_5_ compare VC_i_, VC_u_, VC_e_, and VC_o_ with *V*_REF_. *D*_i_, *D*_u_, *D*_e_, and *D*_o_ become high when VC_i_, VC_u_, VC_e_, and VC_o_ are lower than *V*_REF_. *I*_6_, *I*_7_, and *I*_8_ are the OR gates. *I*_9_ and *I*_10_ with the delay line of *τ* constitute a pulse generator circuit. FF_1_, FF_2_, FF_3_, FF_4_, and FF_5_ are D flip-flop circuits. Output_a_, Output_i_, Output_u_, Output_e_, and Output_o_ are the output signals of five D flip-flops from FF_1_ to FF_5_.

**Figure 5:**
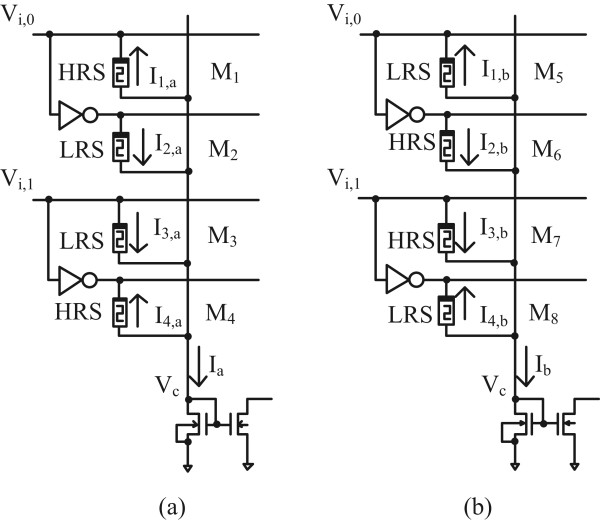
**The forward currents and reverse currents in the matched column (a) and unmatched column (b).***V*_i,0_ = 0 V and *V*_i,1_ = 1 V.

In Figure 4a, we may be concerned that the reverse current through LRS and HRS may degrade the recognition rate. To elaborate on this reverse current more, we assume two cases of memristor crossbar circuit that are matched and unmatched as shown in Figure 5a,b, respectively. In Figure 5a, *V*_i,0_ and *V*_i,1_ are 0 and 1, respectively. These inputs match the stored memristance values of *M*_1_, *M*_2_, *M*_3_, and *M*_4_. Here, HRS means high resistance state and LRS is low resistance state. The current summation of *I*_a_ can be calculated with *I*_a_ = *I*_2,a_ + *I*_3,a_ − *I*_1,a_ − *I*_4,a_. *I*_2,a_ and *I*_3,a_ are the forward currents through *M*_2_ and *M*_3_ that are LRS. *I*_1,a_ and *I*_4,a_ are the reverse currents through *M*_1_ and *M*_4_ that are HRS. In calculating this current summation, *I*_a_ can be expressed simply with *I*_a_ ≈ *I*_2,a_ + *I*_3,a_ because the reverse currents of *I*_1,a_ and *I*_4,a_ are much smaller than the forward currents of *I*_2,a_ and *I*_3,a_. As we know, HRS is much larger than LRS; thus, we can ignore *I*_1,a_ and *I*_4,a_ in calculating *I*_a_. From this explanation, we can know that the reverse current through HRS can affect *I*_a_ very little.

Now, we can consider Figure 5b, where the input voltages of *V*_i,0_ and *V*_i,1_ do not match with the stored memristance of *M*_5_, *M*_6_, *M*_7_, and *M*_8_. The current summation of *I*_b_ in Figure 5b can be expressed with *I*_b_ = *I*_2,b_ + *I*_3,b_ − *I*_1,b_ − *I*_4,b_. Here, *I*_2,b_ and *I*_3,b_ are the forward currents through HRS. *I*_1,b_ and *I*_4,b_ are the reverse currents through LRS. If we compare the matched column's current of *I*_a_ in Figure 5a with the unmatched column's current of *I*_b_, we can be sure that *I*_a_ is much larger than *I*_b_. Thus, we can think that the reverse current does not degrade the recognition rate.

**Figure 6:**
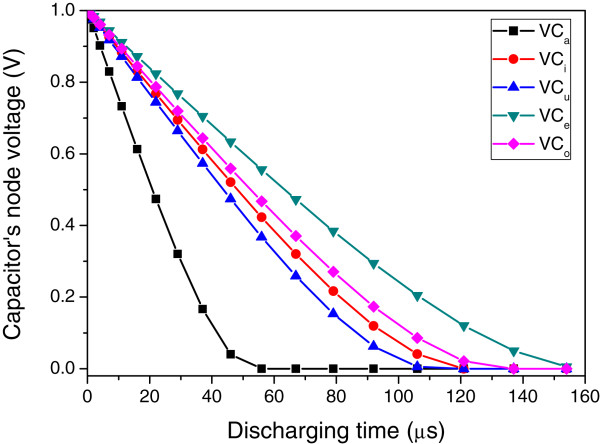
**Capacitor's node voltage with increasing discharging time.** Here, the voice input is ‘a’; thus, VC_a_ falls the fastest among all the node voltages of VC_a_, VC_i_, VC_u_, VC_e_, and VC_o_.

**Figure 7:**
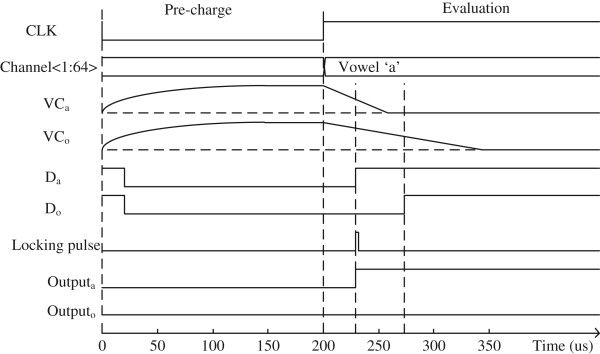
Voltage waveforms of the binary memristor crossbar and winner-take-all circuits.

The simulated waveforms of VC_a_, VC_i_, VC_u_, VC_e_, and VC_o_ are shown in Figure 6. Here, VC_a_ seems to be discharged by GND faster than the other capacitor nodes of VC_i_, VC_u_, VC_e_, and VC_o_. It means that the voice input matches with the vowel ‘a’ better than the other vowels. The timing diagram of important signals in Figure 4a,b is shown in Figure 7. When the CLK signal is low, all the capacitor nodes of VC_a_, VC_i_, VC_u_, VC_e_, and VC_o_ are precharged by *V*_DD_. At this time, VC_a_, VC_i_, VC_u_, VC_e_, and VC_o_ are higher than *V*_REF_; thus, *D*_a_, *D*_i_, *D*_u_, *D*_e_, and *D*_o_ can be low. When the CLK becomes high, five capacitors of *C*_a_, *C*_i_, *C*_u_, *C*_e_, and *C*_o_ can be discharged by *I*_a_, *I*_i_, *I*_u_, *I*_e_, and *I*_o_, respectively. Among *I*_a_, *I*_i_, *I*_u_, *I*_e_, and *I*_o_, if *I*_a_ is the largest amount of current, VC_a_ is discharged by GND faster than VC_i_, VC_u_, VC_e_, and VC_o_. If VC_a_ becomes lower than *V*_REF_, *D*_a_ becomes high. As explained earlier, because VC_a_ is the fastest falling node among the five capacitive nodes, *D*_a_ can also be the fastest rising signal among *D*_a_, *D*_i_, *D*_u_, *D*_e_, and *D*_o_. The fastest rising signal of *D*_a_ can generate the locking pulse that can be used as the clock signal of D flip-flop circuits of FF_1_, FF_2_, FF_3_, FF_4_, and FF_5_. By doing so, we can decide which vowel is the best match to the voice input. The first-rising signal of *D*_a_ makes Output_a_ high, as shown in Figure 7. The other output signals, such as Output_i_, Output_u_, Output_e_, and Output_o_, are prevented from rising from low to high by the locking pulse that is generated by the first-rising signal of *D*_a_.

## Results and discussion

**Figure 8:**
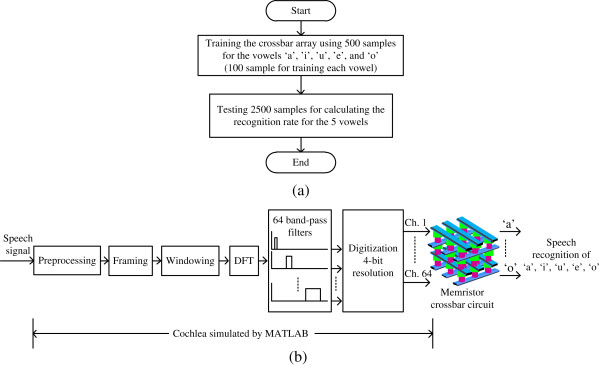
**Training and recalling process of binary memristor crossbar and human cochlea simulation by MATLAB. (a)** Training and recalling of the binary memristor crossbar for recognizing five vowels: ‘a’, ‘i’, ‘u’, ‘e’, and ‘o’, and **(b)** the function of the human cochlea that is simulated by MATLAB software.

In this work, the memristor-CMOS hybrid circuits were simulated by Cadence Spectre software. Here, memristors were modeled by Verilog-A [20,21], and CMOS SPICE parameters were obtained from Samsung's 0.13-μm CMOS technology. The training and recalling process of the memristor crossbar array are shown in Figure 8a. In this paper, we used 100 samples for training a crossbar array to learn the vowel ‘a’. Similarly, we used 400 samples for the crossbar array to learn four vowels: ‘i’, ‘u’, ‘e’, and ‘o’. By the training process, we can find the best memristance values of the crossbar array for maximizing the recognition rate of five vowels: ‘a’, ‘i’, ‘u’, ‘e’, and ‘o’ [18]. The memristance values that are found by the training process were written to the crossbar array circuit by the *V*_DD_/3 write scheme that is known better in mitigating the half-selected cell problem compared to the *V*_DD_/2 write scheme [22].

**Figure 9:**
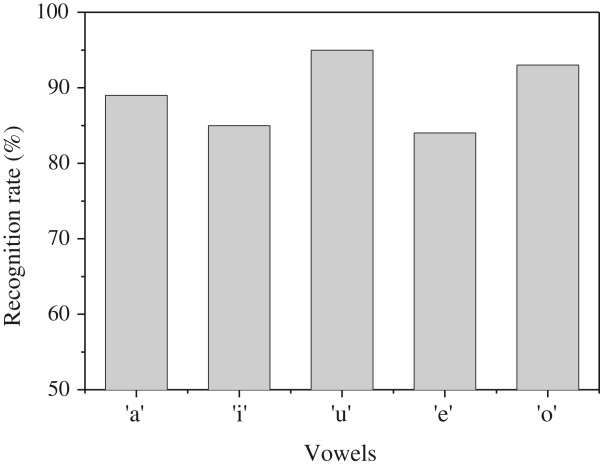
**The simulated recognition rate of binary memristor crossbar for recognizing five vowel: ‘a’, ‘i’, ‘u’, ‘e’, and ‘o.’** Here, the number of tested voices is 2,500.

For the training process, we have to convert the original speech signal to a 4-bit 64-channel digitized signal. In a biological system, the cochlea in the human ear can perform this conversion function. In this paper, we used MATLAB software that performs the same conversion function with the human cochlea. The cochlea function that is simulated by MATLAB software is shown in Figure 8b. The function of the cochlea can be modeled by preprocessing, framing, windowing, discrete Fourier transforming (DFT), band-pass filtering, and digitization [23]. For the digitization process, 64 outputs from 64 band-pass filters are converted to 4-bit binary signals and they are delivered to the rows of the memristor crossbar array. For the band-pass filtering, the nonlinear frequency scale which is known as the mel scale is used [23]. In the mel scale, the frequency scale is linear up to 1,000 Hz and is logarithmic when the input voice has a higher frequency than 1,000 Hz [23].Figure 9 shows the simulation results for the recognition rate of the proposed binary memristor crossbar circuit. In this case, we tested 2,500 input voices for recognizing five different vowels. Each vowel is tested by 500 different voices. The average recognition rate of five different vowels is estimated to be around 89.2%. Among the five vowels, the recognition rate of ‘u’ is the highest at 95.2% while the vowel ‘e’ has the lowest recognition rate, as low as 84%.

**Figure 10:**
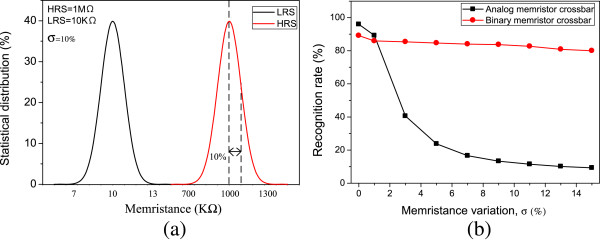
**Statistical distribution of memristance and comparison of recognition rate between analog and binary memristor crossbar. (a)** Statistical distribution of memristance with the standard deviation as much as 10%, and **(b)** comparison of the recognition rate between the analog memristor crossbar and binary memristor crossbar with varying percentage variation in memristance from 0% to 15%.

Figure 10a shows the statistical variation of memristance in HRS and LRS with the standard deviation (=*σ*) of 10%. The statistical variation was obtained by Monte Carlo simulation that was also provided by Cadence software. This statistical simulation is very important because real memristors are susceptible to process variation. To analyze how tolerant the proposed binary memristor crossbar is against the memristance variation, we tested various cases of memristance variation from 0% to 15%. In Figure 10b, we compared the proposed binary memristor crossbar circuit with the analog memristor crossbar one increasing the percentage variation in memristance from 0% to 15%.

When the memristance variation is as low as 0%, the recognition rate of the analog memristor array is higher by 6.8% than the binary memristor array. This is due to the fact that the proposed binary memristor crossbar has a 4-bit resolution; thus, it loses some amount of accuracy compared to the analog memristor crossbar. As the percentage of variation in memristance is increased, the recognition rate of analog memristor crossbar becomes degraded very rapidly. For example, when the percentage variation in memristance becomes 5%, the recognition rate of the analog crossbar is decreased from 96% to 23%. On the contrary, the binary memristor crossbar can keep almost the same amount of recognition rate for five vowels. For a percentage variation as severe as 15%, the analog crossbar shows a recognition rate as low as 9%. However, the binary crossbar still keeps the recognition rate as high as 80%, indicating that it is only degraded by 9.2% compared to the percentage variation of 0%. This strong tolerance of the binary memristor crossbar is due to the fact that the accuracy of the information stored in binary memristors can be little affected by the percentage variation in memristance. Memristance of LRS can still be much smaller and cannot become larger than that of HRS, even though the percentage variation in LRS is very large. This is the reason why the binary memristor crossbar can maintain the recognition rate over 80% regardless of the percentage variation in memristance.

## Conclusions

In this paper, the binary memristor crossbar circuit was proposed for neuromorphic application of speech recognition. Compared with analog memristors that are rare in available materials and need a complicated fabrication process, binary memristors which are based on the filamentary-switching mechanism are found more popularly and easy to be fabricated. Thus, we developed the neuromorphic crossbar circuit using filamentary-switching binary memristors instead of interface-switching analog memristors. The proposed binary memristor crossbar could recognize five vowels with 64 input channels and a 4-bit resolution. The proposed crossbar array was tested by 2,500 speech samples and verified to be able to recognize 89.2% of the total tested samples. Moreover, the recognition rate of the binary memristor crossbar is degraded very little only from 89.2% to 80%, even though the percentage statistical variation in memristance is increased from 0% to 15%. In contrast, the analog memristor crossbar is degraded significantly from 96% to 9% with the same percentage variation in memristance.

## Competing interests

The authors declare that they have no competing interests.

## Authors’ contributions

All authors have contributed to the submitted manuscript of the present work. KSM defined the research topic. SNT and SJH designed the circuit and performed the simulation. KSM wrote the paper. All authors read and approved the submitted manuscript.

## Authors’ information

SNT and SJH are Ph.D. and M.S. students, respectively, who are studying in the School of Electrical Engineering, Kookmin University, Seoul, South Korea. KSM is a professor in the School of Electrical Engineering, Kookmin University, Seoul, South Korea.
